# Avian Haemosporidian Infection in Wildlife Rehabilitation Centres of Portugal: Causes, Consequences, and Genetic Diversity

**DOI:** 10.3390/ani14081216

**Published:** 2024-04-18

**Authors:** João T. Cruz, Luís Madeira de Carvalho, Mariana Ribeiro Ferreira, Carolina Nunes, María Casero, Alfonso Marzal

**Affiliations:** 1 Centre for Interdisciplinary Research in Animal Health (CIISA), Faculty of Veterinary Medicine (FMV-ULisboa), University of Lisbon, 1300-477 Lisbon, Portugal; joaot99fmv@gmail.com (J.T.C.); madeiradecarvalho@fmv.ulisboa.pt (L.M.d.C.); 2 Associate Laboratory for Animal and Veterinary Sciences (AL4AnimalS), 1300-477 Lisbon, Portugal; 3 Centre for Studies and Rehabilitation of Wild Animals of Castelo Branco (CERAS), Quercus ANCN, Rua Tenente Valadim, 17, 6000-284 Castelo Branco, Portugal; ceras@quercus.pt; 4 Wildlife Rehabilitation Centre of Santo André (CRASSA), Quercus ANCN, Moinho Novo, Galiza, 7500-022 Vila Nova de Santo André, Portugal; crassa@quercus.pt; 5 Wildlife Rehabilitation and Investigation Centre of the Ria Formosa (RIAS), Parque Natural da Ria Formosa, 8700-194 Olhão, Portugal; riasaldeia@gmail.com; 6 Department of Anatomy, Cellular Biology and Zoology, University of Extremadura, 06006 Badajoz, Spain; 7 Wildlife Research Group, San Martin National University, Tarapoto 22021, Peru

**Keywords:** avian malaria, wildlife veterinary medicine, avian conservation, treatment duration, *Haemoproteus*, *Leucocytozoon*, *Plasmodium*

## Abstract

**Simple Summary:**

Over thirty percent of bird species are undergoing population declines and are threatened with extinction in Portugal. Several reasons have been proposed to explain this decrease, such as the impact of human activities on natural environments and pathogens affecting the health of wildlife, domestic animals, and humans. Wildlife rescue and rehabilitation centres play an essential role in the conservation of endangered species. Despite wildlife rehabilitation centres providing valuable information on disease prevalence and transmission, the information on haemosporidian infection is still very scarce for birds admitted in these centres. In this study, we discovered new malaria parasites in birds admitted to wildlife rehabilitation centres in Portugal. We also revealed infection in bird species that were previously unknown to be infected with malaria parasites. Birds admitted to rehabilitation due to debilitating disease were more frequently infected with malaria. Furthermore, we demonstrate that the malaria infection extends the required period for medical treatment in these birds, which imposes additional economic costs for the rehabilitation and reduces the survival probabilities of the bird. These findings stress the importance of the study of malaria parasites in wildlife rehabilitation centres, also helping to design protocols and interventions to preserve endangered species.

**Abstract:**

In the last decade, over 40% of bird species in Europe have experienced poor and bad conservation status, with more than 30% of bird species in mainland Portugal threatened with extinction. Along with anthropogenic factors, parasites and pathogens such as avian haemosporidians have been suggested to be responsible for these avian population declines. Wildlife rehabilitation centres play an essential role in species conservation and preservation. Moreover, animals admitted for rehabilitation can provide valuable information regarding transmission and pathogenicity of many diseases that affect wild birds that are rarely sampled in nature. However, reports of haemosporidians in captive birds are still limited. Here, we explored the prevalence and genetic diversity of avian haemosporidians in 89 birds from 29 species admitted to rehabilitation centres in Portugal, showing an overall infection prevalence of 30.3%. The prevalence of infection was higher in Strigiformes and in birds admitted to rehabilitation centres due to debilitating diseases. Remarkably, 30% of the infected bird species have not been found to harbour malaria parasites in preceding studies. We detected 15 different haemosporidian lineages infecting a third of bird species sampled. Notably, 2 out of these 15 detected haemosporidian lineages have not been obtained previously in other studies. Furthermore, we also identified nine new host–parasite interactions representing new host records for these haemosporidian parasites. Finally, our results revealed that birds infected with haemosporidians require longer rehabilitation treatments, which increase the economic costs for rehabilitation and may impair their survival prospects. These findings emphasise the importance of integrating haemosporidian infection considerations into rehabilitation protocols, highlighting the challenges posed by these infections in avian conservation and rehabilitation, including economic and logistical demands.

## 1. Introduction

The global-scale decrease in animal populations represents one of the most dramatic consequences of human impacts on the planet, where 48% of animal species are undergoing population declines [1]. This situation is dramatic for some groups of vertebrates, such as birds, where 5412 out of the 11,162 analysed species (48,5%) face reductions in population size in both temperate and tropical regions [1,2]. For example, the proportion of birds having poor and bad conservation status and the number of avian species threatened with extinction have increased in Europe in the last decade [3,4]. Even though population trends in common bird species in Europe have revealed a change in abundance since the 1980s, the decline is not equally distributed among all countries [5]. In mainland Portugal, there are 404 confirmed wild bird species [6]. In 2022, the conservation status of 287 bird species was evaluated, revealing that 95 of them were classified as threatened with extinction (Vulnerable, Endangered, or Critically Endangered) [7]. This marks an increase from the 88 species of birds classified as threatened in 2005 [8].

Studies analysing long-term data of bird populations have proposed that anthropogenic factors are responsible for these avian population declines. For example, agricultural intensification, in particular, the use of pesticides and fertilisers, is one of the main pressures for invertebrate feeders [5]. Also, the offspring production of migratory and larger-bodied avian species has been negatively affected by the rising temperatures associated with climate change [9]. Moreover, forest degradation has led to broad-scale declines for most forest bird species in Canada [10]. Parasites and pathogens are also major threats facing bird species that can cause rapid declines in wild bird populations. For example, *Trichomonas gallinae* emerged as a novel pathogen of finches in Britain in 2005 and rapidly became epidemic, leading to a decrease in 2007 in breeding populations of greenfinch (*Carduelis chloris*) and chaffinch (*Fringilla coelebs*) by 35% and 21%, respectively [11]. Furthermore, the highly pathogenic avian influenza H5 epidemic has caused the highest number of casualties among wild birds ever recorded in Europe [12].

Avian malaria and related haemosporidian parasites (Apicomplexa; order Haemosporida; *Plasmodium*, *Haemoproteus* and *Leucocytozoon* spp.) represent a highly diverse group of haemoparasites, with over 5100 parasite lineages documented to infect nearly 2300 bird species (MalAvi database Version 2.5.8, 24 October 2023, [13]). They are commonly referred as “avian malaria” due to the malaria-like clinical signs they often cause [14,15]. These parasites are transmitted exclusively by blood-sucking dipteran insects [16]. Culicidae mosquitoes are responsible for the transmission of avian *Plasmodium* parasites, whereas *Haemoproteus* parasites are transmitted by biting midges (Ceratopogonidae) and louse flies (Hippoboscidae), and *Leucocytozoon* are vectored by black flies (Simuliidae) [17]. Although avian haemosporidian parasites were considered as relatively benign for a long time (see review in [18]), they have been reported to negatively affect the host condition [19,20], clutch size [21], reproductive success [19,21,22,23], and lifespan [24], subsequently diminishing host fitness [25,26,27]. The clinical signs and pathology of bird haemosporidian infection include fever, anoxia, tissue necrosis (e.g., liver and spleen), acute anaemia, pneumonia-like symptoms, and excessive enlargement of the spleen and liver that can lead to organ rupture [16]. Moreover, haemosporidian infections can also lead to a reduction in haematocrit levels that may result in death [28,29] and to an increase in stress proteins (heat shock proteins) [30].

Wildlife rehabilitation centres involve the treatment and temporary care of injured, diseased, and displaced indigenous animals, and the subsequent release of healthy animals to appropriate habitats in the wild [31]. In these centres, an adequate treatment and assessment of prognosis is relevant to promote a quick and effective release into the wild. For these reasons, the length of stay in rehabilitation centres (the difference between the date of admission and the date when the stay of the individual in the rehabilitation centre was terminated) has been proposed as an estimator of the cost of the rehabilitation process (cost in staff, food, and medicines) and a useful tool for the evaluation of wildlife rehabilitation centres [32]. However, this parameter is barely reported in studies of wildlife rehabilitation (see [32,33,34] for some exceptions). Furthermore, even though individuals admitted to rehabilitation centres are frequently parasitised by blood parasites [35,36,37], the impact of avian haemosporidian infections on the length of stay in the rehabilitation centres is largely unknown. Moreover, wild birds maintained in captivity are an excellent model for parasite research and provide valuable results for wildlife management conservation of endangered of poorly studied species [36,37]. Important advances in the study of avian haemosporidians, such as the discovery of the life cycle of some avian haemosporidians [16], the disclosure of the negative impact of the parasite infection on the health of their hosts [16,38], or the development of many anti-parasite drugs and chemotherapies [18] have been possible thanks to the use of individuals from zoos and rehabilitation centres. Despite this importance, reports of haemosporidians in captive birds are still scarce, with only 277 avian haemosporidian linages recorded in captive birds, which represents 6% of the identified haemosporidian lineages available in the MalAvi database (Version 2.5.8, October 2023, [13]).

Here, we present a molecular-based study to explore the infection by haemosporidian parasites in birds admitted to wildlife rehabilitation centres in mainland Portugal. Our main objectives were (1) to assess the prevalence and genetic diversity of avian haemosporidians in rescued birds; (2) to analyse the factors explaining variation in haemosporidian infection; and (3) to evaluate the association of the haemosporidian infection and the number of days those wild birds admitted to the rehabilitation centre required medical treatment.

## 2. Materials and Methods

### 2.1. Sample Collection

The study was conducted in three wildlife rehabilitation centres in mainland Portugal: (1) Centre for Studies and Rehabilitation of Wild Animals of Castelo Branco (CERAS) in Castelo Branco (39°49′27.6″ N 7°27′15.1″ W), (2) Wildlife Rehabilitation Centre of Santo André (CRASSA) in Setúbal (38°04′27.0″ N 8°46′56.8″ W), and (3) Wildlife Rehabilitation and Research Centre of Ria Formosa (RIAS) in Faro (37°02′03.1″ N 7°48′45.7″ W).

From November 2022 to May 2023, we collected blood samples from birds admitted to rehabilitation centres upon intake at the centres and from birds already undergoing medical treatment. Throughout that period, 89 wild birds were sampled, belonging to 29 distinct species spanning across 13 taxonomic orders. For each individual, a small blood volume (50 µL) was collected in heparinised microcapillaries by puncturing the brachial vein and stored at 4 °C in 0.5 mL of SET-buffer (0.015 M NaCl, 0.05 M Tris, 0.001 M EDTA, pH 8.0) until molecular analysis. The Eppendorf tubes with blood samples in SET-buffer were stored in the refrigerator at 4 °C until they were sent to the Faculty of Biology of the University of Extremadura, Badajoz, Spain, for further processing and analysis. The body condition of each individual was evaluated upon admission through palpation of pectoral muscle mass and assessment of the prominence of the keel bone, graded on a scale of 1 to 5. A score of 1 indicated extreme emaciation, while a score of 5 indicated overweight condition [39]. The prepatent period is the elapsed time from the inoculation of sporozoites by the vector into the bird until the appearance of blood stages. This period varies from 11–21 days for *Haemoproteus*, approximately five days for *Leucocytozoon*, and possibly as short as five days in the case of *Plasmodium relictum* [16]. Fifty-six birds were sampled at the admission on the rehabilitation centres or before the prepatent period of haemosporidian parasites was over, whereas the remaining 33 individuals were sampled when they were already housed in the centres for longer periods. Hence, for statistical analyses exploring the effect of haemosporidian infection on the required medical treatment for birds admitted to a rehabilitation centre and the factors influencing haemosporidian infection probability, we only considered birds sampled in the centre for less than the corresponding prepatent period of the observed parasite infecting the bird (n = 56), therefore ensuring that all analysed infections were acquired in the wild and not in the wildlife rehabilitation centre. All samples (n = 89) were considered for the identification of parasite lineages and lineage–host interactions.

### 2.2. Sample Processing and Molecular Determination of Haemosporidian Infection

Blood samples were examined using molecular methods to determine the presence of and genetically characterise haemosporidian parasite lineages at the Faculty of Biology of the University of Extremadura. DNA was extracted from all the samples preserved in SET-buffer using a MAGMAX PATHOGEN RNA/DNA KIT (Applied Biosystems™, Waltham, MA, USA, reference: 4462359). The DNA was also quantified, using a NanoDrop microvolume spectrophotometer (Thermo Fisher Scientific™, Waltham, MA, USA) and diluted to 25 ng/µL. The DNA was then stored at −20 °C until further examination. 

For the genetic analysis of haemosporidian infection, a nested-PCR protocol was conducted to amplify a segment of the mitochondrial cytochrome b gene of haemosporidians, as described by Bensch et al. [40] and Hellgren et al. [41]. This technique enables the screening of haemosporidians within the *Plasmodium*, *Haemoproteus*, and *Leucocytozoon* genera [41]. In the first PCR, two general haemosporidian primers, HaemFNI and HaemNR3, were utilised to amplify DNA from all three haemosporidian genera [41]. In the second PCR, a specific set of primers were used for *Haemoproteus* and *Plasmodium* spp. (HaemF and HaemR2) and for *Leucocytozoon* spp. (HaemFL and HaemR3L) [41]. We performed an additional nested PCR, following the procedure of Waldenström et al. [42], to address some methodologic problems found in three samples, namely, the false positives for *Haemoproteus*/*Plasmodium* in samples intensely infected with *Leucocytozoon*. In these additional nested PCRs, we used two specific primers to *Haemoproteus* and *Plasmodium* in the first PCR: HaemNF and HaemNR2. In the second PCR of these samples, we used the same primers as described above (HaemF and HaemR2). 

The PCR mix for all parasites was carried out in a final volume of 25 µL, including 15.9 µL of water, 2.5 µL of Buffer (10× Ex Taq Buffer TaKaRa, Shiga, Japan), 2.5 µL of dNTPs (dNTP Mix TaKaRa, Shiga, Japan), 1 µL of each primer, and 2 µL of DNA. All PCR assays contained 1 negative control (sterilised water) for every 8 samples, and 2 positive controls (samples from infected birds, confirmed by microscopy) for every 24 samples. The PCRs were conducted using a SimpliAmp™ Thermal Cycler (Applied Biosystems™, Foster City, CA, USA), following the thermal profile of Hellgren et al. [41]. The amplification was evaluated by running 2.5 µL of the final PCR on a 2% agarose gel. Haemosporidians detected by a positive amplification were sequenced using the procedures described by Bensch et al. [40]. Sequences were edited and aligned using the program BioEdit [43]. The ‘Basic Local Alignment Search Tool’ (Blast) of GenBank (Accessed 28 July 2023) and the MalAvi database (Version 2.5.8, October 2023, [13]) were used to determine the lineage of detected parasite sequences. Parasites with sequences differing by at least 1 nucleotide from the already described lineages of both databases were considered new evolutionary independent lineages [40].

New lineages (sequences not previously published in GenBank) were also sequenced from the reverse end using the primers HaemR2 or HaemR3L to confirm that they were unique. New lineages were coded following the nomenclature of the MalAvi database [13] and deposited in GenBank under the accession numbers PP457803-PP457804 (Supplementary Table S1). New host–parasite relationships were established by comparison of our results with the public database (MalAvi database Version 2.5.8, October 2023, [13]) showing avian haemosporidian lineages (based on mitochondrial cytochrome b lineages) and host range. A new host record was established when the avian haemosporidian lineage had not been previously reported infecting this bird species, as has been done in previous studies [44,45].

### 2.3. Statistical Analysis

A General Linear Model (GLM) was used to analyse the relationship between body condition, haemosporidian infection (infected vs. uninfected), rehabilitation centre (CERAS, CRASSA and RIAS), and the season of admission (winter, spring, and autumn) on the number of days requiring medical treatment for birds admitted to the rehabilitation centre. The dependent variable was log-transformed to fit a linear model. A logistic regression analysis was used to explore whether avian taxonomic order, body condition, rehabilitation centre (CERAS, CRASSA and RIAS), the season of admission to rehabilitation centres (winter, spring or summer), and the reasons of admission to rehabilitation centres (debilitating disease, trauma, or other causes of admission) influenced haemosporidian infection probability (infected/uninfected). A backward stepwise procedure was used to eliminate all non-significant terms (*p* > 0.05) from our starting maximal model. All analyses were performed using PASW Statistics 22 statistical package for Windows.

## 3. Results

### 3.1. Prevalence and Genetic Diversity of Haemosporidian Parasites

A total of 89 bird individuals belonging to 29 bird species were screened for haemosporidian parasites. Twenty-seven out of the 89 individuals were infected with haemosporidians (overall prevalence = 30.3%). Of these sampled birds, 19.1% were infected with *Haemoproteus*, 13.5% were infected with *Leucocytozoon*, and 1.1% were infected with *Plasmodium*. Additionally, three individuals (3.6% of prevalence) harboured mixed infections with *Leucocytozoon* and *Haemoproteus* (Table 1).

The overall prevalence of haemosporidian infection of the 56 birds sampled at the time of admission to the rehabilitation centres or before the prepatent period of haemosporidian parasites was over was 41.1%—25% prevalence for *Haemoproteus*, with a 16.1% prevalence for *Leucocytozoon*—and 1.8% prevalence for *Plasmodium* (Table 1). 

We detected 15 different parasite lineages infecting 10 out of the 29 avian species sampled (n = 89). Among these, we found nine *Leucocytozoon* lineages infecting seven bird species, five *Haemoproteus* lineages infecting five bird species, and one *Plasmodium* lineage infecting one bird species (Supplementary Table S1). By comparison of genetic diversity of haemosporidian parasites, we showed that two out of the 15 lineages from our study had not been previously recorded in former studies (Supplementary Table S1). Moreover, we found that three out of the nine bird species tested positive for infection in this study had not been previously documented as infected by haemosporidian parasites in molecular studies (Table 1). Furthermore, we also identified nine new host–parasite interactions, which represent new bird host records for these haemosporidian parasites (Supplementary Table S1).

### 3.2. Factors Determining the Length of Medical Treatment

**Figure 1:**
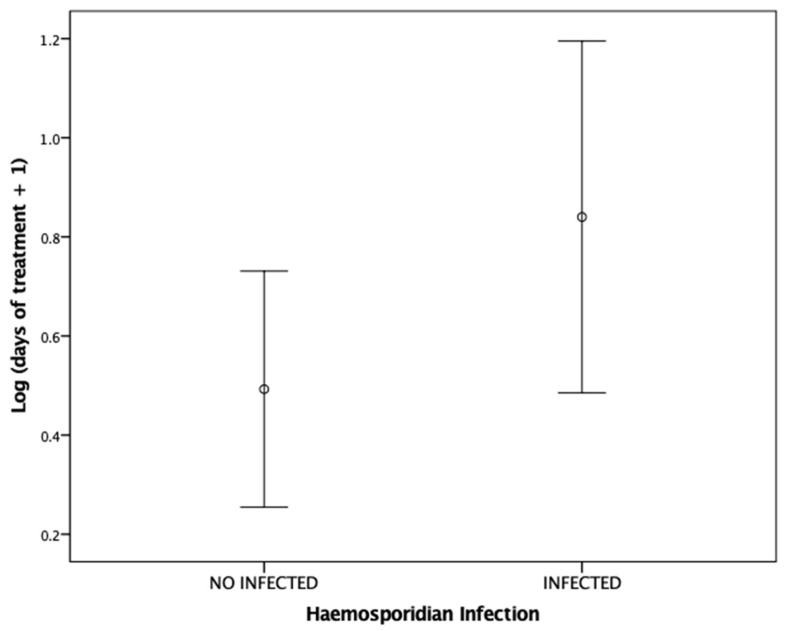
Error bar plots (mean ± 95% CI) showing the number of days (log transformed) that haemosporidian-infected (n = 23) and -uninfected (n = 33) birds admitted to rehabilitation centre required medical treatment.

**Figure 2:**
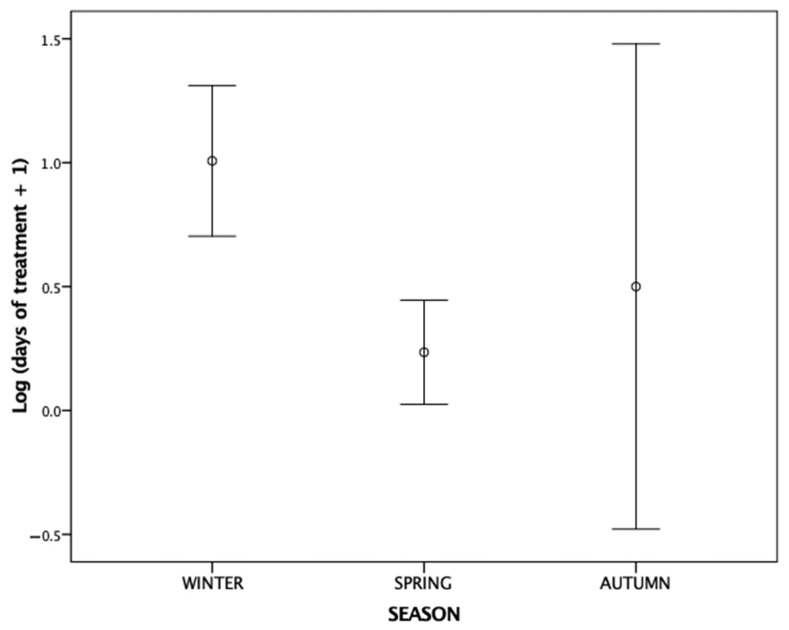
Error bar plots (mean ± 95% CI) showing the number of days (log transformed) requiring medical treatment for birds admitted to rehabilitation centre with respect to the season when they were admitted: winter (n = 28), spring (n = 25), and autumn (n = 3).

The length of medical treatment required by the birds admitted to the rehabilitation centres differed according to the haemosporidian infection and the season when they were admitted (Table 2). Specifically, haemosporidian-infected birds significantly required longer treatments than uninfected individuals (mean days of treatment (SD): infected = 28.22 (56.89); uninfected = 9.85 (16.81)) (Figure 1). Also, birds admitted to rehabilitation centres during winter (n = 28) received medical care for longer time periods than birds admitted during spring (n = 25) or autumn (n = 3) (mean days of treatment (SD): winter = 30.43 (51.55); spring = 4.24 (11.98); autumn = 6.50 (11.67)) (Figure 2).

### 3.3. Factors Determining Haemosporidian Infection

We also analysed haemosporidian infection in relation to body condition, avian taxonomic order, the rehabilitation centre where birds were admitted, the season of admission to rehabilitation centres, and the reasons for admission to rehabilitation centres. Avian taxonomic order significantly explained variation in haemosporidian infection (Table 3). Notably, six out of seven (85.7%) of Strigiformes showed haemosporidian infection. Also, 40.6% of Charadriiformes were infected with haemosporidian parasites, whereas only 20% of birds belonging to the order Accipitriformes showed haemosporidian infection (Table 1). Remarkably, the order Strigiformes was also the only order having double infections (*Leucocytozoon* and *Haemoproteus*). In addition, the prevalence of haemosporidian infection also varied with the reasons for admission to rehabilitation centres (Table 3). Birds admitted due to debilitating diseases had a significantly higher prevalence of haemosporidian infection (52.2%, n = 23) compared to cases of admission due to physical trauma (27.6%, n = 31), with the remaining two individuals admitted for undetermined reasons.

## 4. Discussion

Over a third of the approximately 500 bird species living in Europe are threatened or have a poor conservation status [4]. In mainland Portugal, more than 30% of bird populations have been recently categorised as threatened with extinctions [7]. Hence, preserving and restoring avian populations is one of the cornerstones of EU biodiversity policy. Wildlife rehabilitation is an undervalued and potentially useful tool for stabilising some declining populations and could be targeted to support in situ interventions [47]. In this line, rehabilitation centres and other captive breeding facilities play an essential role in species conservation and preservation. Here, we analysed the prevalence and genetic diversity of avian haemosporidians in birds admitted to rehabilitation centres in Portugal to explore the factors explaining variation in haemosporidian infection and to evaluate features influencing the length of required treatment. Our main findings were: (i) about 40% of birds were infected with haemosporidians, also showing a great genetic diversity of haemosporidian lineages and a high number of new host–parasite associations; (ii) birds infected with haemosporidians required longer medical treatments during their stay in the rehabilitation centres; and (iii) the prevalence of avian haemosporidian infection was higher in Strigiformes and in those admitted to rehabilitation centres due to debilitating diseases. Next, we will discuss these results in detail. 

### 4.1. Prevalence and Genetic Diversity of Haemosporidian Parasites 

We showed an overall haemosporidian prevalence of 41% in the wild birds sampled. This probability of infection is similar to those found in recent studies using molecular methodologies to analyse haemosporidian infections in birds admitted to rehabilitation centres. For example, Nourani et al. [48] examined the infection with haemosporidian parasites in captive raptors in two rehabilitation facilities in North and Northeast Iran, determining an overall prevalence of 36%. Likewise, Pornpanom et al. [49] reported a haemosporidian prevalence of 34% in 12 owl species in a raptor rehabilitation unit in Thailand. Also, Gomes et al. [35] explored the occurrence of blood parasites in wild birds from a wildlife rehabilitation centre in Central Portugal, revealing that 48% of sampled birds were positive for haemosporidians. 

Wildlife rehabilitation likely generates millions of animal records annually worldwide [50]. Yet, despite an increasing acknowledgment of the usefulness of wildlife rehabilitation centre data, it remains a prevailing tendency to underutilise this valuable source of information [51]. Moreover, rehabilitation centres provide an excellent opportunity to explore the host range, genetic diversity, and geographic distribution of haemosporidian infections in wild birds which are rarely sampled in nature [52,53]. For example, Gomes et al. [35] have recently described the first occurrence of *Leucocytozoon* sp. in the booted eagle *Hieraaetus pennatus*, the short-toed snake eagle *Circaetus gallicus*, and the European honey buzzard *Pernis apivorus*, and *P. relictum* in the European honey buzzard. Also, Pornpanom et al. [49] reported 17 new lineages of haemosporidian parasites in owls from Southern Asia. Here, we detected 15 haemosporidian lineages infecting a third of bird species sampled. Importantly, we compared our sequences with those in the MalAvi database (Version 2.5.8, October 2023, [13]) and discovered that two out of these 15 detected haemosporidian lineages had not been obtained previously in other studies. Moreover, of these 15 identified haemosporidian lineages, only five (ATNO1, CIAE02, STAL2, GAGLA05, and LINN1) had previously been reported in Portugal, with STRURA03, COCOR02, and STAL3 being also first documented in the Iberian Peninsula, and STAL5 representing the first report outside of Turkey. Furthermore, 30% of the infected bird species had not been found to harbour malaria parasites in preceding studies. Such numbers of newly discovered lineages and the new records of bird hosts infected with blood parasites suggest that the diversity of avian haemosporidians infecting some species of Strigiformes, Charadriiformes, Columbiformes, Pelecaniformes, and Accipitriformes has been insufficiently investigated.

In addition, our analyses also revealed nine new bird–parasite interactions, thus identifying new host records for these haemosporidian parasites. These new bird–haemosporidian associations are made up of the two newly described haemosporidian lineages (ARCIN01 and LARFUS01), plus seven parasite lineages previously identified as infecting alternative hosts. Some of these new host–parasite associations are worthy of being underlined. First, we found a *Leucocytozoon* parasite (GenBank acc. Number OL897562) infecting *Bubo bubo* that has been previously identified in pooled samples of the blackfly *Simulium meridionale* captured throughout Mississippi, USA [54]. Second, *Streptopelia decaocto*, an avian host that had not been reported as infected by haemosporidians in previous studies, was found infected by *Leucocytozoon* ATNO1, a parasite lineage that had been previously recorded exclusively infecting the little owl （*Athene noctua*) (MalAvi database Version 2.5.8, October 2023, [13]). Finally, the other detected haemosporidian lineages had been identified in related hosts in previous studies. For example, the *Haemoproteus* lineage (GenBank acc. Number ON950078) infecting *L. fuscus* and *L. michahellis* was recently described in other larid hosts [55], the *Leucocytozoon* lineage MILVUS02 is commonly found in other Accipitriformes [56,57], and *Plasmodium matutinum* LINN1 is a generalist haemosporidian lineage found infecting species of a wide range of avian orders, including Charadriiformes (MalAvi database Version 2.5.8, October 2023, [13]). The new diversity records on host–parasite interactions provided in this study will be valuable for detecting host range and transmission areas of haemosporidian parasites, and will improve our knowledge of the mechanisms of adaptation of avian haemosporidians to new hosts. However, we should be cautious in the interpretation of these new records because these new host–parasite interactions may not fully determine the competence of these avian hosts supporting development of infective stages (gametocytes in avian hosts) that can reach a new host [58,59]. In this line, some haemosporidian parasites may infect avian hosts without completing their full life cycle, hence leading to abortive development in these dead-end hosts. Because the amplification of parasite DNA by PCR does not distinguish gametocytes from asexual parasite stages [60], further studies examining blood smears to detect the presence of gametocytes circulating in peripheral blood are needed to complement our findings and determine the competence of these avian hosts to transmit these parasites. Moreover, microscopy is a fast and cheap methodology that allows the quantification of parasitemias and to detect mixed infections, which are sometimes difficult to find with molecular screening. Furthermore, although low-intensity avian malaria infections could be difficult to detect solely by microscopic examination of blood smears, the combined use of PCR and traditional microscopy could be especially relevant to connect genetic lineages with morphospecies and describe new species of haemosporidians [60].

### 4.2. Factors Determining the Length of Medical Treatment

The process of wildlife rescue and rehabilitation encompasses the rescue, treatment, and care of injured, sick, or orphaned native animals, with the goal of their release into their natural habitat or a more suitable environment [31]. However, a prolonged period in captivity can result in loss of survival skills in wildlife [61]. In this line, Cope et al. [62] have recently conducted a global systematic review and meta-analysis evaluating the factors influencing the success of wildlife rehabilitation, concluding that shorter periods of rehabilitation enhance the probabilities of survival of released animals after the treatments. Wild birds admitted to rehabilitation centres worldwide are frequently parasitized by haemosporidians [35,63,64], although whether haemosporidian infection may extend the rehabilitation period in wild birds remains largely unknown. Our findings revealed that birds infected with haemosporidian required longer periods of medical treatment than non-infected birds, which may affect survival up to release or survival post-release [62]. Therefore, the initial diagnosis of haemosporidian infections in wild birds admitted to rehabilitation centres becomes crucial for an early assignation of a correct anti-malaria treatment that could minimise their length of stay in the centre and thus enhance their survival prospects.

Rehabilitation centres are often self-funded or heavily subsidising their own rehabilitation work [47,65], hence facing constraints due to insufficient funding, staff availability, and access to appropriate veterinary care [66]. Beyond the mentioned increased fitness benefits to the wildlife of shorter rehabilitation periods, a timely assessment and treatment of haemosporidian infection can also have an economic impact on the rehabilitation centre, as it may promote the effective use of limited resources. In this line, the length of medical treatment has been proposed as an indicator of resource usage [67]. The daily cost per animal in a wildlife rehabilitation centre has been estimated at EUR 0.19 [32]. According to the mean values of the length of stay of haemosporidian-infected and non-infected birds from our study (28.22 days and 9.85 days, respectively), the average expenses of rehabilitation of a non-infected bird can be estimated at approximately EUR 1.9, whereas these costs rise to EUR 5.4 for an infected bird, representing an additional cost of EUR 3.5 per animal.

Our results also show a significant increase in the duration of medical treatment of the birds admitted to rehabilitation centres during the winter months. This finding could be attributed to the reluctance to release rescued birds into the wild during winter, when seasonal environmental conditions are less favourable [68], consequently prolonging their stay in the centres. Alternatively, because metabolic disorders are associated with high recovery times in wild birds [32], the extended period of rehabilitation during winter can also be explained by the higher metabolic costs associated with thermogenesis in winter [69,70]. The large variation in days of treatment showed in Figure 2 for autumn can be explained because birds admitted to rehabilitation during that season are released either quickly, before winter, or held over winter [68]. However, these data should be interpreted with caution because of the low number of sampled birds admitted to rehabilitation centres during that season (n = 3).

### 4.3. Factors Determining Haemosporidian Infection

Our findings showed differences in the prevalence of infections among avian orders in birds brought to wildlife centres in Portugal, where birds from the orders Strigiformes and Charadriiformes showed the highest probabilities of being infected. Several factors have been proposed to explain why some bird species are prone to becoming infected with haemosporidians. For example, colonial bird species (such as most species of Charadriiformes), or those with larger body sizes or prolonged stays of their nestlings on the nests, usually show a high prevalence of infections [16]. Also, these differences in haemosporidian prevalence have been suggested to be determined by vector preferences [71] or host behaviour characteristics [27,71,72]. For example, the higher prevalence of haemosporidians in owls compared to diurnal birds of prey has been well documented [73,74,75,76], and it has been attributed to two primary factors. First, Strigiformes have nocturnal behaviour, which coincides with the crepuscular or nocturnal hours in which mosquito vector species perform host-seeking behaviour [74,77]. Second, their preference for concealed and shaded perches during the day, as well as their nesting sites, may expose Strigiformes more frequently to a variety of haemosporidian vector species [78].

Finally, we showed that the prevalence of infection was higher in birds admitted to the rehabilitation centres due to debilitating diseases than in birds admitted for other causes. Several experimental studies have demonstrated that haemosporidian parasites may impair the physiology of their avian hosts, provoking anaemia [79], the blockage of brain capillaries [80], a diminished body condition, a decrease in fat reserves and atrophy of pectoral muscles [81], and reduced haematocrit [82]. All these negative effects may explain the observed association between infection and debilitating disease in birds. Alternatively, a poor body condition, inadequate nutritional status, or heightened stress levels in birds may compromise their immune system [83,84], and thus increase the likelihood of haemosporidian infection of these debilitated birds [16].

## 5. Conclusions

This study assessed the prevalence and genetic diversity of haemosporidian parasites in birds at wildlife rehabilitation centres in mainland Portugal, also analysing their effect on the required rehabilitation period and the factors explaining their infection. We have revealed newly discovered parasite lineages and new records of bird hosts infected with blood parasites, thus confirming that the diversity of avian haemosporidians is still insufficiently investigated in some avian species. In addition, these findings are also relevant because host-switching of blood parasites is relatively frequent among birds housed in zoos and rehabilitation centres, provoking fatal infections [85,86]. We have also identified that Strigiformes and birds admitted to rehabilitation due to debilitating disease showed the highest probabilities of being infected with haemosporidians, highlighting the reciprocal relationship between debilitating state and blood parasite infection. Moreover, our study sheds light on the largely unknown impact of avian haemosporidian infections on the length of stay in the rehabilitation centres. Haemosporidian-infected individuals required nearly three times more days in veterinary care compared to non-infected counterparts, impairing their survival prospects and exacerbating resource constraints in wildlife rehabilitation centres. These findings emphasise the need for integrating analyses of haemosporidian infection into diagnostic and treatment protocols, also highlighting the importance of blood sampling the same day of admittance to the rehabilitation centre. Moreover, the seasonal variations observed in the length of veterinary treatment needed, particularly during winter months, stress the importance of adaptive management strategies that account for seasonal fluctuations in rehabilitation demands. In light of our results, some additional recommendations are indicated for further studies exploring factors influencing the haemosporidian infection, such as larger sample sizes and incorporating data from summer infections. Also, repeated blood sampling of individuals beyond the day of admittance to the rehabilitation centre would allow assessment of the effectiveness of treatment and monitoring of whether birds are infected during their stay in the centre. Because the study of blood parasites is also relevant to control parasite infections in birds before translocation or liberation [87], the insights gained from this study have significant implications for avian conservation and wildlife rehabilitation efforts. By highlighting the challenges posed by haemosporidian infections in avian conservation and rehabilitation, this study emphasises the importance of future research endeavours aimed at enhancing our understanding of avian health and guiding conservation strategies.

## Figures and Tables

**Table 1:** Number of individuals uninfected, and infected with *Haemoproteus* (H), *Plasmodium* (P), and *Leucocytozoon* (L), per bird species. The information on migratory behaviour from each species (Migratory (M), Resident (R), or Undetermined (U)) obtained from [46] is also shown. Numbers in brackets represent the number of individuals for each bird species that were sampled at the time of admission on the rehabilitation centres or before the prepatent period of haemosporidian parasites was over. An asterisk (*) after bird species denotes that these bird species were not previously documented to be infected by haemosporidians (according to (MalAvi database Version 2.5.8, October 2023, [13]), where the symbol **^$^** in *Haemoproteus* infection (H) indicates mixed infection with *Leucocytozoon*.

Bird Species	Bird Order	Migratory	Uninfected	H	P	L	Total
*Accipiter gentilis*	Accipitriformes	R	0	0	0	1 (0)	1 (0)
*Aegypius monachus*	Accipitriformes	R	3 (0)	0	0	0	3 (0)
*Alca torda*	Charadriiformes	M	1 (1)	0	0	0	1 (1)
*Anas platyrhynchos*	Anseriformes	R	1 (1)	0	0	0	1 (1)
*Ardea cinerea*	Pelecaniformes	M	0	0	0	1 (1)	1 (1)
*Asio flammeus*	Strigiformes	M	1 (0)	0	0	0	1 (0)
*Bubo bubo*	Strigiformes	R	0	1 ^$^ (1 ^$^)	0	4 (3)	4 (3)
*Bubulcus ibis*	Pelecaniformes	R	1 (1)	0	0	0	1 (1)
*Burhinus oedicnemus*	Charadriiformes	R	1 (1)	0	0	0	1 (1)
*Buteo buteo*	Accipitriformes	R	1 (0)	0	0	1 (1)	2 (1)
*Ciconia ciconia*	Ciconiiformes	R	10 (2)	0	0	0	10 (2)
*Falco tinnunculus*	Falconiformes	R	2 (0)	0	0	0	2 (0)
*Fratercula arctica*	Charadriiformes	M	1 (1)	0	0	0	1 (1)
*Gallinula chloropus*	Gruiformes	R	1 (1)	0	0	0	1 (1)
*Garrulus glandarius*	Passeriformes	R	0	1 ^$^ (0)	0	1 (0)	1 (0)
*Gyps fulvus*	Accipitriformes	R	2 (2)	0	0	0	2 (2)
Larus fuscus *	Charadriiformes	M	6 (4)	8 (8)	0	0	14 (12)
Larus michahelis *	Charadriiformes	R	17 (10)	5 (4)	1 (1)	0	23 (15)
*Larus* sp.	Charadriiformes	U	2 (2)	0	0	0	2 (2)
*Milvus migrans*	Accipitriformes	M	1 (0)	0	0	0	1 (0)
*Milvus milvus*	Accipitriformes	R	3 (2)	0	0	0	3 (2)
*Morus bassanus*	Suliformes	M	1 (1)	0	0	0	1 (1)
*Phalacrocorax carbo*	Suliformes	M	1 (1)	0	0	0	1 (1)
Streptopelia decaocto *	Columbiformes	R	0	0	0	1 (1)	1 (1)
*Strix aluco*	Strigiformes	R	1 (0)	1 ^$^ (1 ^$^)	0	3 (3)	4 (3)
*Sturnus unicolor*	Passeriformes	R	1 (0)	0	0	0	1 (0)
*Tachybaptus rufficolis*	Podicipediformes	R	1 (0)	0	0	0	1 (0)
*Tyto alba*	Strigiformes	R	2 (1)	0	0	0	2 (1)
*Upupa epops*	Bucerotiformes	R	2 (2)	0	0	0	2 (2)
Total			63 (33)	16 (14)	1 (1)	12 (9)	89 (56)

**Table 2:** Factors explaining variation in the number of days those wild birds admitted to the rehabilitation centre required treatment. A General lineal model was used with body condition, haemosporidian infection, rehabilitation centre (locality), and the season of admission to rehabilitation centres as predictor variables. Sample size was 56 individuals. Significant factors are highlighted in bold.

Variable	Type III SS	d.f.	F	*p*
Body condition	0.017	1	0.038	0.846
Haemosporidian infection	2.172	1	4.747	**0.034**
Rehabilitation centre	0.010	1	0.021	0.884
Season	6.112	1	13.358	**0.001**

**Table 3:** Factors explaining variation in the probability of haemosporidian infection. A backward stepwise procedure was used in a logistic regression analysis with avian taxonomic order, body condition, rehabilitation centre (locality), the season of admission to rehabilitation centres, and the reasons for admission to rehabilitation centres as predictor variables. Only independent variables selected by the stepwise procedure are listed. Significant factors are highlighted in bold.

Variable	*B*	S. E.	Wald	d.f.	*p*	Exp (B)
Avian taxonomic order	0.180	0.091	0.943	1	**0.047**	1.197
Reason for admission	−1.256	0.524	5.741	1	**0.017**	0.285
Constant	0.442	0.868	0.259	1	0.611	1.555

## Data Availability

The authors confirm that the data supporting the findings of this study are available within the article and in Supplementary Table S1.

## Supplementary Materials

The following supporting information can be downloaded at: https://www.mdpi.com/article/10.3390/ani14081216/s1, Table S1: MalAvi parasite lineages, Parasite, genus (H *Haemoproteus*, P *Plasmodium*, L *Leucocytozoon*), GenBank accession numbers, recorded host in this study (Host), and alternative hosts and alternative location in which parasite lineages were previously recorded.
