# Supplementary material for: Avian Haemosporidian Infection in Wildlife Rehabilitation Centres of Portugal: Causes, Consequences, and Genetic Diversity
Source: Animals (Basel). 2024 Apr 18;14(8):1216. doi: 10.3390/ani14081216 (PMC11047687; doi:10.3390/ani14081216)
Supplement: Supplementary file 1 [file animals-14-01216-s001.zip › animals-2960686-supplementary.pdf]

animals

MDPI

Supplementary Table 1. MalAvi parasite lineages, Parasite, genus (H *Haemoproteus*, P *Plasmodium*, L *Leucocytozoon*), GenBank accession numbers, recorded host in this study (Host), and alternative hosts and alternative location in which parasite lineages were previously recorded. Asterisk (*) in parasite lineage indicates new haemosporidian lineages not recorded in previous studies, where symbol $ represents new host record for this haemosporidian lineage and where symbol # represents new geographical record for this haemosporidian lineage (according to MalAvi database, Version 2.5.8, October 2023, [13]).

MalAvi lineage	Parasite	Genus	GenBank #	Host	Alternative host	Alternative location
Not defined #	*Leucocytozoon* sp.	L	OL897562	*Bubo bubo*$		
Not defined #	*Haemoproteus* sp.	H	ON950078	*Larus fuscus*$; *Larus michahellis*$		
ARCIN01* #	*Leucocytozoon* sp.	L	PP457803	*Ardea cinerea*$		
ATNO1	*Leucocytozoon* sp..	L	KJ488699	*Streptopelia decaocto*$	*Athene noctua*	Europe (Portugal), Africa (Morocco)
BUBO01 #	*Leucocytozoon danilewskyi*	L	MK330142	*Bubo bubo*	*Bubo bubo*	Europe (Spain)
CIAE02	*Leucocytozoon* sp.	L	MK330160	*Buteo buteo*	Falconiformes, Gruiformes, Charadriiformes, Piciformes, Strigiformes, Ciconiiformes, Cuculiformes, Coraciiformes, Phoenicopteriformes	Europe (Spain, Germany, Poland, Portugal, Sweden, Austria), Asia (Philippines, Turkey, Russia, Mongolia, Japan, China, Thailand), Africa (South Africa)
COCOR02 #	*Leucocytozoon* sp.	L	JX867111	*Garrulus glandarius*	*Garrulus glandarius*, *Corvus corax*	Europe (Bulgaria, Slovakia, Germany)
GAGLA05	*Haemoproteus* sp.	H	KJ488735	*Garrulus glandarius*	*Garrulus glandarius*, *Corvus corax*	Europe (Bulgaria, Portugal), Africa (Morocco)
LARFUS01* #	*Haemoproteus* sp.	H	PP547804	*Larus fuscus*$; *Larus michahelis*$		
LINN1	*Plasmodium matutinum*	P	MK330156	*Larus michahellis*$	Passeriformes, Apterygiformes, Sphenisciformes, Gruiformes, Strigiformes, Charadriiformes, Falconiformes	Europe (Austria, Hungary, Sweden, United Kingdom, Portugal, Spain, Czech Republic, Italy, Germany, Switzerland, Slovakia, Poland), Oceania (New Zealand), Asia (Japan) North America (USA)

MILVUS02 #	*Leucocytozoon* sp.	L	JN164717	*Accipiter gentilis*$	*Buteo buteo, Buteo lagopus, Milvus milvus, Haliaeetus albicilla*	Europe (Austria, Spain)
STAL2	*Haemoproteus syrnii*	H	KJ488773	*Strix aluco*	*Strix aluco, Strix nebulosa, Strix uralensis, Bubo bubo*	Europe (Portugal, France, Germany), Africa (Morocco)
STAL3 #	*Leucocytozoon* sp.	L	MK652258	*Strix aluco*	*Strix aluco*	Europe (Austria)
STAL5 #	*Leucocytozoon* sp.	L	KC876042	*Strix aluco*	*Strix aluco*	Asia (Turkey)
STRURA03 #	*Haemoproteus* sp.	H	KJ488826	*Bubo bubo*	*Bubo scandiacus, Bubo bubo, Strix nebulosa, Strix uralensis*	Europe (Austria, France, Switzerland)
